# Effects of website-based risk communication of radio-frequency electromagnetic fields on general public

**DOI:** 10.3389/fpubh.2024.1438986

**Published:** 2024-09-04

**Authors:** Sachiko Yamaguchi-Sekino, Kazuhisa Kamegai, Miwa Ikuyo, Masao Taki, Teruo Onishi, Soichi Watanabe

**Affiliations:** National Institute of Information and Communications Technology, Koganei, Japan

**Keywords:** radio-frequency electromagnetic field, concerns of adverse health effects, risk communication, risk perception, objective knowledge

## Abstract

**Background:**

Radio-frequency electromagnetic fields (RF-EMFs) are utilized in communications and appliances and are indispensable in daily life. However, some people have concerns about the adverse health effects of RF-EMFs; therefore, effective risk communication (RC) is needed in this field.

**Objective:**

In this study, we investigate public attitudes towards RF-EMFs and examine the impact of RC via a website on these attitudes and objective knowledge.

**Methods:**

Three web surveys were conducted over 10 weeks with the same participants. The questionnaires were conducted at three different time points with 5-week intervals: baseline survey (T1), RC evaluation survey (T2), and follow-up survey (T3). Participants of T2 were randomly recruited from among those of T1, and participants of T3 were randomly selected from among the T2 respondents. Approximately half of the respondents in each of T2 and T3 were assigned to the control group. Twelve items regarding attitudes toward RF-EMFs and objective knowledge were evaluated in all surveys (T1–T3). After removing low-engagement data, the number of valid answers was 782 in T3. Differences between T1 and T2 (Sub T1-T2) and T1 and T3 (Sub T1-T3) were analyzed. Participant selection was randomized and the authors were blind to this selection until analysis.

**Results:**

Four clusters were identified: Cluster 1 (Non-anxious, 25.0%), Cluster 2 (Anxious, 16.0%), Cluster 3 (Low-interest, 40.5%), and Cluster 4 (High-interest, 18.5%). A decrease in subjective RF-EMF exposure levels was noted in Cluster 2 immediately after website viewing. Temporary increases and decreases in health concerns about RF-EMF usage activities were observed in Clusters 1 and 2, respectively, immediately after viewing. Clusters 1 and 3 showed a temporal decrease in needs for RF-EMF usage activities at T2 but it returned to the baseline level 5 weeks later. Cluster 4 was less responsive to the risk communication. Subanalysis stratified by gender and age showed fluctuations in responses, especially in Clusters 1 and 2.

**Conclusion:**

We demonstrate the effectiveness of RF-EMF risk communication via websites, particularly for Cluster 2. The results of this study showed that offering objective and comprehensible information through a website can significantly reduce concerns and perceived risks related to RF-EMFs.

## 1 Introduction

Radio-frequency electromagnetic fields (RF-EMFs) are defined by the World Health Organization as ranging from 10 MHz to 300 GHz (1) and by the International Commission on Non-Ionizing Radiation Protection as ranging from 100 kHz to 300 GHz (2). RF-EMFs are utilized in communications and home appliances, and they have become essential in everyday life. The biological effects of EMFs have been scientifically established to be short-term, such as nerve stimulation only (100 kHz–10 MHz) (3), the combination of nerve stimulation and body heating (100 kHz–10 MHz) (4), and body heating only (> 10 MHz) (4) at very high intensities; therefore, body heating is a considerable effect of RF-EMFs. The international protection guidelines for these short-term effects have been published (3, 4). However, regarding long-term effects such as carcinogenesis, despite many studies that have been conducted, scientifically substantiated harmful effects have not been reported (5–7), but continued research is required. This safety information is provided by international organizations (5) and various countries through various means, but a certain number of people are still concerned about the adverse health effects of RF-EMFs, and continuous risk communication (RC) is needed.

The development of the internet and social media may expose the general public to research on the health effects of RF-EMFs. However, while experts can point out the limitations of the research and aspects that should be interpreted with caution (8), the general public may overlook these nuances and encounter information that could lead to unnecessary increases in risk perception. Therefore, appropriate institutions must engage in RC with the general public regarding various types of RF-EMF information.

Many studies showed the impact of information presented (9–17), such as precautionary measures (10–14) and objective exposure levels (15–17), on risk perception. Regarding information content, there have been reports showing an increase in EMF risk perception as a result of presenting precautionary measures (10–12), although other studies showed no change (13, 14).

Moreover, so far, sharing the measurement results has been known to change the risk perception or other factors related to RF-EMFs (15–17). Ramirez-Vazquez et al. reported that sharing the RF-EMF exposure results of personal exposimeters can reduce risk perception (9). Other studies showed that the presentation of objective RF-EMF exposure levels did not affect risk perception, but increased trust in the measures for protection against RF-EMF exposure (16, 17). Although the full impact awaits clarification, these reports underscore the utility of objective RF-EMF exposure levels in risk communication (15–17). In particular, monitoring actual RF-EMF exposure levels and presenting this information has become the focus of risk communication in recent years (18–20). Our group has also started research on the measurement of RF-EMF exposure levels in daily life (21–24) in parallel with the implementation of risk communication using measurement results.

However, for the information receiver, in general, it is necessary to use audience segmentation and provide information in accordance with the segments (25–27). The need for similar actions is recognized in RF-EMF risk communication. The importance of information receiver characteristics was reported (28). Boehmert et al. conducted a systematic review of health risk communication regarding EMFs from wireless technologies (28). In this systematic review, they not only examined the main impacts of communication on risk perception but also studied how the characteristics of the information receiver can significantly influence the formation of that perception. However, there are no studies on identifying segments in accordance with their attitudes toward RF-EMFs in the general public or examining how risk communication works for each segment. Moreover, no studies have followed up on changes in risk perception over time after risk communication (e.g., 1 month later).

Considering the current lack of studies on segmentation examples in the field of RF-EMF risk communication, in this study, we investigate the types of cluster in the general public by classifying respondents on the basis of their attitudes toward RF-EMFs and examine how web-based risk communication affects attitudes and objective knowledge of RF-EMFs in each cluster.

## 2 Methods

### 2.1 Website for risk communication of RF-EMFs

Since our RF-EMF exposure level monitoring can be considered a means of presenting objective RF-EMF exposure levels, such a presentation of information could potentially influence people’s perception of RF-EMFs. Therefore, we created a pilot website that includes monitoring results and basic information about the physics and safety of RF-EMFs so that people can understand them and implement risk communication for the general public. The website developed was handled as a pilot website because we are planning to modify it in the future using the results of this study. Thus only a limited number of respondents were allowed to access the website.

Before the study, our group implemented a pilot survey to investigate the public needs and concerns about adverse health effects caused by RF-EMFs through a web survey of 5,625 respondents from July to August 2022 (data not shown). For example, respondents replied incorrectly (15.2%) or “no idea” (65.1%) to questions on the objective knowledge of the basic physics of RF-EMFs (e.g., Q: “When the human body is exposed to RF-EMFs, part of the energy is absorbed and converted into heat.”). 35.6% of respondents answered that they had absolutely “no idea” of their perception of RF-EMFs in daily life (subjective RF-EMF exposure levels). Through this survey, we found a lack of basic knowledge of RF-EMFs, a lack of information on objectively measured RF-EMF levels, and concerns about the adverse health effects of RF-EMFs in the general public. Therefore, our group developed a pilot website covering these issues.

Our monitoring project is a national initiative of the Ministry of Internal Affairs and Communications of Japan aimed at providing substantiated information to the public regarding RF-EMF levels in their surroundings. These monitoring methods range widely from spot measurements that intensively measure specific locations, mobile measurements using personal measurement devices, fixed-point measurements for long-term measurements, and nationwide measurements using car-mounted measurement devices. The monitoring periods also varied, including instances and long-term periods (daily measurements for several months or more). In this report, we present the monitoring results, including a comparison of the spot measurements of RF-EMFs from a mobile phone base station taken at the same locations in 2011 and 2021 (100 measurement locations within a 1 km square area) (21), measurements around broadcasting stations using spot methods (24), and household wireless LAN measurements using measurement devices (23). All measurement results were significantly below the guideline values set in Japan, with levels as much as several hundred times lower than the maximum level.

The contents of our pilot website include (1) an introduction to our RF-EMF exposure level monitoring project (21–24), (2) explanations of RF-EMF exposure levels in daily life based on our measurement results (from mobile phone base stations, broadcasting stations, and wireless LAN stations), (3) the basics of RF-EMF physics [two levels for beginners and intermediate (the level between beginners and experts)], (4) the use of RF-EMFs in daily life, (5) explanations of the adverse health effects of RF-EMFs and guidelines (two levels for beginners and intermediate), (6) frequently asked questions (25 items), and (7) an index.

The expected targets for beginner-level content were respondents with little knowledge of or less interest in RF-EMFs. Therefore, a Q&A conversation style was used for better understanding. The content for the intermediate level was composed of text referring to scientific papers to provide more information than for the beginner level. The expected targets for the intermediate-level content were respondents with a certain level of knowledge or interest in RF-EMFs.

The pilot website underwent reviews by experts to check the validity of its content. Before the survey, the website was shown to a small group of the general public to gather feedback on its difficulty, readability, and impression. However, since it is a pilot website, the design has not been reviewed by specialists. In the web survey, screenshots were provided for (1), (2), and (5) (beginners’ level) before answering questions. In addition, participants were permitted to browse the website freely during the survey.

### 2.2 Study design

**Figure 1:**
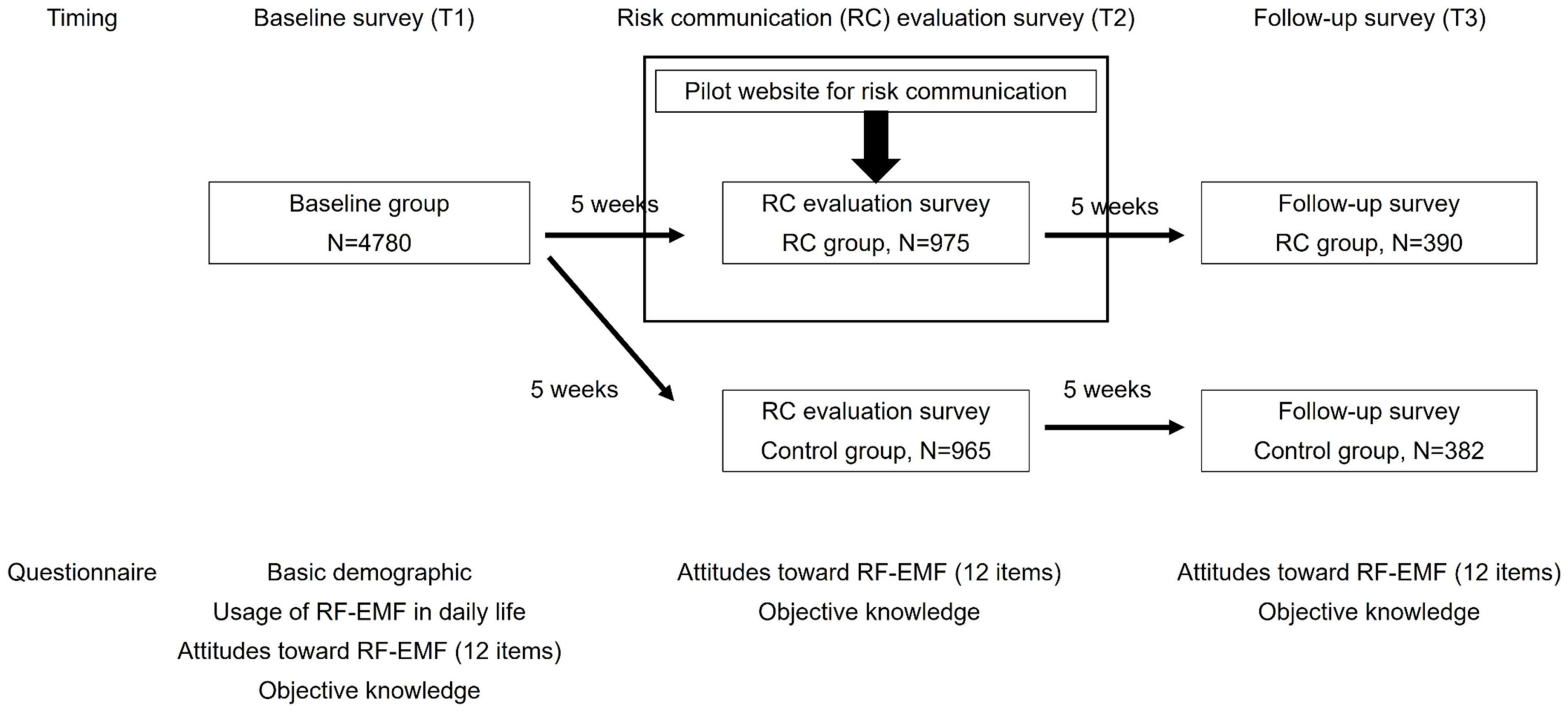
Study protocol.

A web survey was conducted three times with the same subjects (Figure 1). The first survey (Baseline study, Timing 1: T1) was conducted in October 2023 with 5,007 participants to investigate the general public’s attitudes toward RF-EMFs. Study participants were recruited using a commercial panel and targeted Japanese or Japanese residents, with adjustments made to ensure that the age and gender distributions matched the national census.

The second survey was aimed at evaluating the website-based RC (RC evaluation survey, Timing 2: T2). This survey was conducted 5 weeks after T1 to investigate the impact of viewing our constructed website on attitudes toward RF-EMFs. This duration was set exploratively, considering the lack of research on the persistence of the impact of RF-EMF risk communication on awareness, making the T2–T3 period 5 weeks. For T2, respondents were recruited from among T1 participants and divided into two groups: the risk communication group (RC group) and the control group. Only the RC group had access to the website. In T2, screenshots were provided for some content on the website (see Section 2.1) before answering the questions and these were provided only to the RC group. Additionally, participants in the RC group were permitted to browse the website freely during the survey. All participants answered the same questionnaire items as those in T1. In the RC group, participants answered the questionnaire immediately after viewing the website, and the control group responded to the questionnaire without viewing the website.

The third survey (Follow-up survey, Timing 3: T3) was conducted 5 weeks after T2. In T3, respondents were recruited from among T2 participants, and their attitudes toward RF-EMFs were resurveyed in the same manner as in T2.

### 2.3 Participant selection and quality control measures

Study participants were recruited using a commercial panel under randomized and blinded conditions. The questionnaire was targeted at Japanese individuals or residents who could understand the Japanese language because the questionnaires were written in Japanese. In this study, quota sampling was applied to match age and gender distributions to those of the national census. In our case, the supplier sets a limit for the number of respondents based on age and gender distribution in the national census (nationally representative sample based on national census data). However, because of the limited number of registered respondents in the commercial panel, we manually set the number of respondents over age 70 (100 in T1, 20 in T2, and 8 in T3) under randomized and blinded conditions. In T2 and T3, participants of the two groups (RC and control groups) were obtained in the same manner; gender and age distributions were adjusted to those of the national census. The call for participation was automatically closed when a sufficient number of respondents were collected for each of the age and gender groups.

We estimated that the participation rate in follow-up studies would be 40% for T2 and T3. Therefore, the minimum number of respondents was set at 2000 and 800 for T2 and T3, respectively. For T1, 5,007 respondents completed the baseline survey. The respondents in T1 received a call for the T2 survey 5 weeks later, and 2000 participated in the survey. During sample collection, respondents were randomly assigned to either the RC group or the control group, and the 1,000 respondents each in the RC group and the control group completed the survey. The respondents in T2 received a call for the T3 survey 5 weeks later. We collected 401 respondents from among RC group members and 401 from among control group members in T2.

For quality control measures, the survey company eliminated the answers with extremely quick responses and responses with a string of random characters in the open-ended question. Moreover, data from T1 (*N* = 5,007) were screened by excluding the respondents who declared their gender to be “other” (*n* = 7) owing to the small sample size. Additionally, respondents with the following combination of answers were eliminated to exclude those with low engagement in the questionnaire (*n* = 220): “Q: What is the signal strength of your mobile phone” with “A: no idea,” and those who responded “no idea” to all 10 questions regarding objective knowledge of RF-EMFs. After data screening, the final numbers of valid responses were 4,780 for T1, 1,940 (RC group, 965; control group, 975) for T2, and 774 (RC group: 384 and control group: 390) for T3.

### 2.4 Questionnaire survey

On the basis of the results of the pilot survey (see Section 2.1), the questionnaire was designed to exploratively investigate the following five categories concerning RF-EMFs: (1) awareness, (2) perception of exposure (subjective RF-EMF exposure levels), (3) anxiety (including health concerns about RF-EMFs, concern about 5G, and risk perception regarding the carcinogenicity of RF-EMFs), (4) recognition of benefits of 5G, and (5) subjective knowledge. Objective knowledge was also selected to examine the difference between subjective and objective knowledge.

Additionally, regarding sources of exposure, eight sources and six usage behaviors were selected, including microwaves, which were previously associated with high levels of anxiety, as well as other sources such as mobile phone terminals (4G/5G), mobile phone base stations (4G/5G), broadcast waves, wireless LAN, and Bluetooth. The designed questionnaire was reviewed by experts.

The contents of the questionnaires used in T1–T3 are shown in Supplementary Table 1. In T1, we asked about basic demographics, the use of RF-EMFs in daily life (not used in this part for analysis), and the following 12 items on attitudes toward RF-EMFs: (1) awareness of RF-EMFs in daily life, (2) subjective RF-EMF exposure levels (8 sources), (3) confidence in subjective RF-EMF exposure levels (8 sources), (4) concerns about adverse health effects of RF-EMF exposure (8 sources), (5) need for RF-EMF usage activities (6 activities, e.g., watching TV or using a microwave), (6) concerns about adverse health effects of RF-EMF usage activities (6 activities), (7) awareness of the advantages of 5G, (8) awareness of concerns about 5G, (9) subjective knowledge about RF-EMFs, (10) carcinogenic risk perception (RF-EMFs: mobile phones), (11) carcinogenic risk perception (extremely low-frequency EMFs: ELF-EMFs) in terms of the EMFs, and (12) carcinogenic risk perception (5 risk sources excluding RF-EMFs and ELF-EMFs, UV, passive smoking, red meat, pickles, and coffee). Additionally, objective knowledge about RF-EMFs was examined. For objective knowledge about RF-EMFs in T2 and T3, 5 out of 10 questions were related to the contents viewed on the website. Therefore, only the RC group was expected to retain or increase the number of correct answers compared with those of T1 and the control group.

### 2.5 Statistical analyses

#### 2.5.1 Validity and reliability assessment of the questionnaire

Content validity was reviewed and discussed by experts. To assess intrarater reliability, the intraclass correlation coefficient (ICC) was calculated for the 12 items of attitude toward RF-EMFs among T1, T2, and T3 responses of the control group.

Cronbach’s alpha is used to assess internal consistency. On the basis of the strategy of questionnaire construction (see Section 2.4), four items regarding anxiety about RF-EMFs were selected to assess the internal consistency. The following four items were used for analysis: (1) concerns about adverse health effects of RF-EMF exposure (sum of 8 sources), (2) concerns about adverse health effects of RF-EMF usage activities (sum of 6 activities), (3) awareness of concerns about 5G, and (4) carcinogenic risk perception (RF-EMFs: mobile phone). The scores on these items in the baseline survey were standardized (*z*-score), and Cronbach’s alpha was calculated.

Additionally, a preliminary and exploratory factor analysis was conducted. For the issue of normality, the generalized least-squares method and Promax rotation were used in the factor analysis to determine a four-factor structure, and Cronbach’s alpha was calculated for the subscales of the resulting factors. The four-factor structure was determined on the basis of the size of eigenvalues. Subjective exposure levels were excluded from the analysis as they did not exhibit sufficient factor loadings.

#### 2.5.2 Cluster analysis

Cluster analysis was performed using the responses from T1 (*N* = 4,780) in a nonhierarchical cluster analysis. Nonhierarchical cluster analysis was used because of its high reliability in classifying large samples. The 12 items regarding attitudes toward RF-EMFs, except for objective knowledge, were used as variables. We excluded objective knowledge from the cluster analysis to focus on only attitudes toward RF-EMFs in cluster classification. For preprocessing, all variables were scaled so that high scores indicated a greater degree of agreement with the survey items, followed by the standardization of the score of each variable (*z*-score). A nonhierarchical cluster analysis (*k*-means) was performed with a maximum of 100 iterations. In this study, we did not reevaluate the cluster classification of the respondents in T2 or T3 because we focused on clarifying the effects of RC on respondents based on the initial classification in T1. The cluster of each respondent was identical throughout T1–T3. The clusters were named on the basis of the score for each item regarding attitudes toward RF-EMFs. Differences in cluster distribution among T1–T3 were tested using the chi-square test (Supplementary Figure 1). No statistical significance was observed in the cluster distribution at each survey timing.

#### 2.5.3 Analysis between follow-up RC group (*n* = 384) and follow-up control group (*n* = 390)

For the 12 items regarding attitudes toward RF-EMFs and objective knowledge, the Shapiro–Wilk test was conducted as a test of normality, and since the results were *p* < 0.05, nonparametric tests were applied. To analyze the effects of RC on attitudes toward RF-EMFs, the differences between T1 and T2 (Sub T1-T2) and between T1 and T3 (Sub T1-T3) were calculated and used for analysis. Differences in scores were analyzed using the Mann–Whitney U-test. Effect size was also calculated using *Z* score and sample size. Differences in basic demographics between the RC and control groups were analyzed using the chi-square test.

#### 2.5.4 Analysis among 4 clusters in follow-up RC group (*n* = 384) and follow-up control group (*n* = 390)

For the 12 items regarding attitudes toward RF-EMFs, the Shapiro–Wilk test was conducted as a test of normality, and since the results were *p* < 0.05, nonparametric tests were applied. The main analysis was performed to examine the effect of viewing the website in each cluster. The differences between T1 and T2 (Sub T1-T2) and between T1 and T3 (Sub T1-T3) were calculated and used for analysis. The statistical difference between two groups was analyzed using the Mann–Whitney U-test. Effect size was also calculated using *Z* score and sample size. The subjective exposure level, that is, the total sum for eight sources of exposure, was used for analysis. For the needs of RF-EMF usage activities and concerns of adverse health effects related to RF-EMF usage activities, the total sum of the six behaviors was used as data. For the analysis of differences among the 4 clusters, the Kruskal–Wallis test and post-hoc tests (intergroup analyses) using the Bonferroni-adjusted Mann–Whitney U-test were performed.

#### 2.5.5 Subgroup analysis in 4 clusters

Subgroup analyses were performed on T3 respondents. They were divided into two groups on the basis of gender and age (male or female, and age 20–49 or over 50). Because of the small population of young people (age 20–39) in clusters 2 and 3, we decided to divide the group into two: the younger generation (age 20–49) and the older generation (age over 50). The changes in subjective RF-EMF levels and concerns for adverse health effects in RF-EMF usage activities immediately after RC (Sub T1-T2) were analyzed. These two factors were chosen because statistically significant differences in several parameters were detected. The Mann–Whitney U-test was performed to analyze the differences in gender and age within each cluster. Effect size was also calculated using Z score and sample size. Intercluster differences were analyzed using the Kruskal–Wallis test followed by the Bonferroni-adjusted Mann–Whitney U-test.

#### 2.5.6 Analysis of objective knowledge

Q1–Q5 in T2 and T3 were questions regarding objective knowledge of general RF-EMFs, whereas Q6–Q10 were related to the contents of the risk communication website. Therefore, to assess the RC group’s understanding of the website, the number of correct answers and the number of “no idea” responses from the RC and control groups in T2 and T3 were compared. The number of correct answers and the number of “no idea” responses to Q1–Q5 and Q6–Q10 in T2 and T3 for each group were calculated, and the differences between the RC and control groups were tested by the Mann–Whitney U-test. The effect size was calculated using the *z*-score and sample size.

## 3 Results

### 3.1 Validity and reliability assessment of the questionnaire

ICC (1, 3) was between 0.686–0.866, indicating the good intrarater reliability of this questionnaire. Cronbach’s alpha of four items of anxiety regarding RF-EMFs (health anxiety and perceived carcinogenic risk) was 0.825, indicating sufficient internal consistency in terms of anxiety.

As a result of the factor analysis (Supplementary Table 2), the four factors were named as follows: Factor 1, Risk perception; Factor 2, Anxiety; Factor 3, Knowledge and awareness; and Factor 4, Benefit recognition. Cronbach’s alpha values for the four factors were Factor 1 = 0.80, Factor 2 = 0.77, Factor 3 = 0.62, and Factor 4 = 0.52.

### 3.2 Changes in attitudes toward RF-EMFs between RC and control groups

Table 1 shows the characteristics of participants in the RC and control groups. The basic demographic of RC and control groups was males (51.0% in the RC group and 50.5% in the control group): age of 20–29, 16.1% (RC group) and 16.7% (control group); age of 30–39, 16.7% (RC group) and 17.2% (control group); age of 40–49, 22.7% (RC group) and 22.6% (control group); age of 50–59, 23.4% (RC group) and 22.1% (control group); and age over 60, 21.1% (RC group) and 21.0% (control group). No significant differences were observed between the RC and control groups in terms of basic demographics and attitudes toward RF-EMFs in T1.

**Table 1:** Demographic information of the control and RC groups.

Baselin survey (T1)			Control (*n* = 390)		RC group (*n* = 384)		*p*
Category	Variables	Evaluation	*n*	%	*n*	%	
Socio-demographic information	Sex	Male	197	50.5%	196	51.0%	0.89
		Female	193	49.5%	188	49.0%	
	Age (years)	20–29	65	16.7%	62	16.1%	1.00
		30–39	67	17.2%	64	16.7%	
		40–49	88	22.6%	87	22.7%	
		50–59	88	22.6%	90	23.4%	
		> = 60	82	21.0%	81	21.1%	
	Living area in Japan	Area 1–13	–	–	–	–	1.00
	Status of employment	Employed	260	66.7%	264	68.8%	0.54
		Not employed	130	33.3%	120	31.3%	
	Education (general)	Grade 1 (junior high or high school)	102	26.2%	91	23.7%	0.60
		Grade 2 (technical college)	69	17.7%	77	20.1%	
		Grade 3 (undergraduate or graduate school)	219	56.2%	216	56.3%	
	Education (RF-EMFs)	No	346	88.7%	341	88.8%	1.00
		Yes	44	11.3%	43	11.2%	
	Number of family member	1	69	17.7%	59	15.4%	0.94
		2	125	32.1%	129	33.6%	
		3	103	26.4%	103	26.8%	
		4	70	17.9%	69	18.0%	
		> = 5	23	5.9%	24	6.3%	
	Live with family member <=15	No	70	21.8%	52	16.0%	0.07
		Yes	251	78.2%	273	84.0%	
	Family income	Grade 1(< 3 million JPY)	73	18.7%	67	17.4%	0.99
		Grade 2 (3–6 million JPY)	124	31.8%	124	32.3%	
		Grade 3 (6–9 million JPY)	82	21.0%	83	21.6%	
		Grade 4 (> = 9 million JPY)	64	16.4%	62	16.1%	
		No answer	47	12.1%	48	12.5%	

		Control (*n* = 390) median (25th percentile to 75th percentile)	RC group (*n* = 384) median (25th persentile to 75th persentile)	*p*
Attitudes toward RF-EMFs and objective knowledge	Awareness of RF-EMFs in daily life	2.0 (1.0 – 2.0)	2.0 (1.0 – 2.0)	0.43
	Subjective RF-EMF exposure levels (sum of 8 sources)	24.0 (21.0 – 29.0)	25.0 (21.3 – 30.0)	0.16
	Confidence of subjective RF-EMF exposure levels (sum of 8 sources)	16.0 (8.0 – 20.0)	16.0 (8.0 – 20.0)	0.92
	Concerns about adverse health effects of RF-EMF exposures (sum of 8 sources)	16.0 (8.0 – 17.0)	16.0 (9.0 – 18.0)	0.39
	Needs for RF-EMF usage activities (sum of 6 activities)	18.0 (16.0 – 21.0)	18.0 (16.0 – 21.0)	0.92
	Concerns about adverse health effects of RF-EMF usage activities (sum of 6 activities)	12.0 (6.0 – 13.0)	12.0 (6.0 – 13.0)	0.82
	Awareness of the advantages of 5G	1.0 (0.0 – 2.0)	1.0 (0.0 – 2.0)	0.88
	Awareness of concerns about 5G	0.0 (0.0 – 0.0)	0.0 (0.0 – 0.0)	0.91
	Subjective knowledge	1.0 (1.0 – 2.0)	1.0 (1.0 – 2.0)	0.13
	Carcinogenic risk perception (RF-EMFs: mobile phone)	3.0 (2.0 – 4.0)	4.0 (3.0 – 4.0)	0.24
	Carcinogenic risk perception (ELF-EMFs: power lines)	4.0 (3.0 – 5.0)	4.0 (3.0 – 5.0)	0.33
	Carcinogenic risk perception (sum of other 5 sources)	18.0 (16.0 – 21.0)	19.0 (16.0 – 21.0)	0.92
	Objective knowledge	6.0 (4.0 – 8.0)	6.0 (4.0 – 8.0)	0.71

Scores of attitudes toward RF-EMFs and objective knowledge from the baseline survey are also listed in this table. The chi-square test was applied to the analysis of socio-demographic information and the Mann–Whitney U-test to the analysis of attitudes toward RF-EMFs and objective knowledge.

To analyze the effects of RC on attitudes toward RF-EMFs, the differences between T1 and T2 (Sub T1-T2) and between T1 and T3 (Sub T1-T3) were calculated and used for analysis. Data were expressed as median (25 percentile to 75 percentile), and “*r*” represents the effect size derived using *Z* score and sample size.

When comparing the attitudes toward RF-EMFs between the RC and control groups in T2 and T3, various changes in the scores of items were observed immediately after website viewing (Sub T1-T2) in the RC group (Table 2). In the RC group, the level of awareness of RF-EMFs significantly increased and scores of subjective RF-EMF exposure levels significantly decreased (Mann–Whitney U-test, *p* = 0.01 and *p* = 0.03, *r* = 0.13 and 0.11, respectively). However, the need for RF-EMF usage activities and the awareness of the advantages of 5G decreased (Mann–Whitney U-test, *p* = 0.01 and *p* < 0.001, *r* = 0.14 and 0.15). Subjective knowledge significantly increased in the RC group (Mann–Whitney U-test, *p* < 0.001, *r* = 0.23). For objective knowledge, the number of correct answers decreased in the control group in T2, but no such decrease was observed in the RC group (Mann–Whitney U-test, *p* = 0.01, *r* = 0.13). This is because five out of the 10 questions in T2 and T3 were related to website content so only the RC group was expected to answer these questions correctly. Five weeks after website viewing (Sub T1-T3), there were no significant changes in attitudes toward RF-EMFs.

**Table 2:** Changes in attitudes toward RF-EMFs from T1-T2 (Sub T1-T2) and T1-T3 (Sub T1-T3): control group vs. RC group.

	Sub T1-T2					Sub T1-T3				
Variable	Control (*n* = 390) median (25th percentile to 75th percentile)	RC group (*n* = 384) median (25th percentile to 75th percentile)	*p*	*r*	Diection of RC	Control (*n* = 390) median (25th percentile to 75th percentile)	RC group (*n* = 384) median (25th percentile to 75th percentile)	*p*	*r*	Diection of RC
Awareness of RF-EMFs in daily life	0.0 (0.0 – 0.0)	0.0 (0.0 – 1.0)	**0.01**	0.13	↑	0.0 (−1.0 – 0.3)	0.0 (−1.0 – 0.0)	0.93	0.00	–
Subjective RF-EMF exposure levels (sum of 8 sources)	0.0 (−3.3 – 3.0)	−1.0 (−6.0 – 2.0)	**0.03**	0.11	↓	0.0 (−4.0 – 4.0)	0.0 (−5.0 – 3.0)	0.27	0.06	
Confidence of subjective RF-EMF exposure levels (sum of 8 sources)	0.0 (0.0 – 6.0)	0.0 (0.0 – 7.0)	0.68	0.02	–	0.0 (−0.3 – 8.0)	0.0 (0.0 – 7.0)	0.46	0.04	
Concerns about adverse health effects of RF-EMF exposures (sum of 8 sources)	0.0 (−1.0 – 2.0)	0.0 (−1.0 – 3.0)	0.06	0.10	–	0.0 (−1.0 – 2.0)	0.0 (−1.0 – 1.0)	0.71	0.02	
Needs for RF-EMF usage activities (sum of 6 activities)	0.0 (−2.0 – 2.0)	0.0 (−2.8 – 1.0)	**0.01**	0.14	↓	0.0 (−1.0 – 2.0)	0.0 (−1.0 – 2.0)	0.05	0.10	
Concerns about adverse health effects of RF-EMF usage activities (sum of 6 activities)	0.0 (−1.0 – 1.0)	0.0 (−0.8 – 2.0)	0.21	0.06	–	0.0 (0.0 – 1.0)	0.0 (−1.0 – 0.0)	0.08	0.09	
Awareness of the advantages of 5G	0.0 (0.0 – 1.0)	0.0 (0.0 – 0.0)	**0.00**	0.15	↓	0.0 (0.0 – 1.0)	0.0 (0.0 – 1.0)	0.65	0.02	
Awareness of concerns about 5G	0.0 (0.0 – 0.0)	0.0 (0.0 – 0.0)	0.40	0.04	–	0.0 (0.0 – 0.0)	0.0 (0.0 – 0.0)	0.85	0.01	
Subjective knowledge	0.0 (0.0 – 0.0)	0.0 (0.0 – 1.0)	**0.00**	0.23	↑	0.0 (0.0 – 0.0)	0.0 (0.0 – 0.0)	0.08	0.09	
Carcinogenic risk perception (RF-EMFs: mobile phone)	0.0 (−1.0 – 1.0)	0.0 (−1.0 – 1.0)	0.23	0.06	–	0.0 (−1.0 – 1.0)	0.0 (−1.0 – 1.0)	0.32	0.05	
Carcinogenic risk perception (ELF-EMFs: power lines)	0.0 (−1.0 – 1.0)	0.0 (−1.0 – 0.0)	0.14	0.07	–	0.0 (−1.0 – 1.0)	0.0 (−1.0 – 1.0)	0.99	0.00	
Carcinogenic risk perception (sum of other 5 sources)	1.0 (−1.3 – 3.0)	0.0 (−1.0 – 3.0)	0.63	0.02	–	0.0 (−1.3 – 3.0)	1.0 (−1.0 – 3.0)	0.92	0.01	
Objective knowledge	−1.0 (−3.0 – 0.0)	−1.0 (−2.0 – 1.0)	**0.01**	0.13	↑	−2.0 (−4.0 – 0.0)	−2.0 (−3.0 – 0.0)	0.27	0.06	

The Mann–Whitney U-test was applied to the analysis. R, effect size; Bold, statistically significant. ↑ Represents the increased effect and ↓ Represents the decreased effect. The “-” represents no statistical change between RC and control group.

### 3.3 Classification of clusters

Attitudes toward RF-EMFs were classified in accordance with the cluster analysis of the baseline survey and named on the basis of the levels of anxiety and interest in RF-EMFs. The participants were clustered into Cluster 1, Non-anxious group (25.0%); Cluster 2, Anxious group (18.5%); Cluster 3, Low-interest group (40.5%); and Cluster 4, High-interest group (19.9%). Table 3 shows the basic demographics and the score for each of the items related to attitudes toward RF-EMFs in T1 for each cluster. Additionally, the cluster distribution was almost identical from T1 to T3 (Supplementary Figure 1, N.S., chi-square test).

**Table 3:** Characteristics of clusters.

Socio-demographic information	All (*N* = 4,780)	Cluster 1 (Non-anxious, *n* = 1,197)	Cluster 2 (Anxious, *n* = 764)	Cluster 3 (Low-interest, *n* = 1937)	Cluster 4 (High-interest, *n* = 882)
Sex/male	51.1%	54.6%	39.5%	40.6%	79.3%
Age	20–39: 33.4% 40–59: 45.0% > = 60: 21.7%	Young-middle age 20–39: 47.6% 40–59: 39.5% > = 60: 12.9%	Middle-senior age 20–39: 23.8% 40–59: 51.2% > = 60: 25.0%	Middle-senior age 20–39: 27.3% 40–59: 46.8% > = 60: 26.0%	All age 20–39: 35.8% 40–59: 43.2% > = 60: 21.1%
Status of employment/employeed	67.3%	66.1%	64.8%	64.0%	78.6%
Education (general)/University	53.1%	51.0%	53.3%	48.9%	64.7%
Education (RF-EMFs:)/specialist	11.7%	5.3%	14.0%	3.9%	35.6%
Number of family member	1 person: 20.0% 2 persons: 30.7% 3–5 persons: 49.3%	1 person: 22.6% 2 persons: 26.1% 3–5 persons: 51.3%	1 person: 16.8% 2 persons: 32.3% 3–5 persons: 50.9%	1 person: 18.7% 2 persons: 33.9% 3–5 persons: 47.3%	1 person: 22.1% 2 persons: 28.5% 3–5 persons: 49.4%
Live with family member <=15/yes	19.7%	17.6%	20.9%	18.7%	24.0%
Family income	Grade 1–2: 50.0% grade 3–4: 35.5% no answer: 14.4%	Low-average grade 1–2: 52.0% grade 3–4: 33.3% no answer: 14.6%	Low-average grade 1–2: 52.3% grade 3–4: 35.0% no answer: 12.8%	Average grade 1–2: 49.0% grade 3–4: 33.1% no answer: 17.8%	Average-high grade 1–2: 47.5% grade 3–4: 44.5% no answer: 7.9%
Use of RF-EMF utilizing devices	–	Average	Average	Inactive	Active

Attitudes toward RF-EMFs and objective knowledge	All (*N* = 4,780)	Cluster 1 (Non-anxious, *n* = 1,197)	Cluster 2 (Anxious, *n* = 764)	Cluster 3 (Low-interest, *n* = 1937)	Cluster 4 (High-interest, *n* = 882)
	Median (25th percentile to 75th percentile)				
Awareness of RF-EMFs in daily life	2.0 (1.0 – 2.0)	1.0 (1.0 – 2.0)	2.0 (2.0 – 3.0)	2.0 (1.0 – 2.0)	2.0 (2.0 – 3.0)
Subjective RF-EMF exposure levels (sum of 8 sources)	24.0 (22.0 – 29.8)	24.0 (19.0 – 28.0)	29.0 (24.0 – 32.0)	24.0 (21.0 – 28.0)	25.0 (23.0 – 30.0)
Confidence of subjective RF-EMF exposure levels (sum of 8 sources)	16.0 (9.0 – 20.0)	11.0 (8.0 – 16.0)	18.0 (16.0 – 24.0)	16.0 (8.0 – 16.0)	22.0 (16.0 – 24.0)
Concerns about adverse health effects of RF-EMF exposures (sum of 8 sources)	16.0 (9.0 – 18.0)	8.0 (8.0 – 11.0)	24.0 (21.0 – 25.0)	16.0 (16.0 – 17.0)	16.0 (12.0 – 17.0)
Needs for RF-EMF usage activities (sum of 6 activities)	18.0 (16.0 – 21.0)	19.0 (17.0 – 21.0)	18.0 (17.0 – 21.0)	18.0 (16.0 – 19.0)	19.0 (17.0 – 22.0)
Concerns about adverse health effects of RF-EMF usage activities (sum of 6 activities)	12.0 (6.0 – 14.0)	6.0 (6.0 – 7.0)	18.0 (15.0 – 19.0)	12.0 (12.0 – 13.0)	12.0 (8.0 – 13.0)
Awareness of the advantages of 5G	1.0 (0.0 – 2.0)	1.0 (0.0 – 2.0)	1.0 (0.0 – 2.0)	0.0 (0.0 – 1.0)	2.0 (1.0 – 3.0)
Awareness of concerns about 5G	0.0 (0.0 – 0.0)	0.0 (0.0 – 0.0)	1.0 (0.0 – 2.0)	0.0 (0.0 – 0.0)	0.0 (0.0 – 0.0)
Subjective knowledge	1.0 (1.0 – 2.0)	1.0 (1.0 – 1.0)	1.0 (1.0 – 2.0)	1.0 (1.0 – 1.0)	2.0 (2.0 – 3.0)
Carcinogenic risk perception (RF-EMFs: mobile phone)	4.0 (3.0 – 4.0)	2.0 (1.0 – 3.0)	5.0 (4.0 – 6.0)	4.0 (3.0 – 4.0)	3.0 (2.0 – 4.0)
Carcinogenic risk perception (ELF-EMFs: power lines)	4.0 (3.0 – 5.0)	2.0 (2.0 – 3.0)	5.0 (5.0 – 6.0)	4.0 (4.0 – 5.0)	4.0 (3.0 – 5.0)
Carcinogenic risk perception (sum of other 5 sources)	19.0 (16.0 – 21.0)	16.0 (14.0 – 18.0)	21.0 (19.0 – 24.0)	19.0 (17.0 – 21.0)	18.0 (16.0 – 21.0)
Objective knowledge	6.0 (3.0 – 8.0)	6.0 (3.0 – 8.0)	6.0 (4.0 – 8.0)	5.0 (2.0 – 7.0)	7.0 (5.0 – 9.0)

The characteristics of Clusters 1 and 2 were differences in “concerns about adverse health effects,” which were detectable in “Concerns about adverse health effects from RF-EMF exposures (sum of 8 sources),” “Concerns about adverse health effects of RF-EMF usage activities (sum of 6 activities),” and carcinogenic risk perceptions. On the basis of this feature, we named Clusters 1 and 2 the “Non-anxious group” and “Anxious group,” respectively. On the other hand, the characteristics of Clusters 3 and 4 were found in “Awareness of the advantages of 5G” and “Objective knowledge,” respectively. Considering the differences in scores of these items, we named Clusters 3 and 4 the “Low-interest group” and “High-interest group,” respectively.

Cluster 1 (Non-anxious group) had slightly more males and a higher proportion of young to middle-aged individuals. The basic demographics of Cluster 1 were males (54.6%): ages of 20–29, 23.2%; ages of 30–39, 24.4%; ages of 40–49, 20.8%; ages of 50–59, 18.7%; and age over 60, 12.9%. There were slightly more unemployed individuals (33.9%) and a lower proportion of individuals with a technical college degree or higher education [17.5% (technical college) /51.0% (university, graduate school)]. The family compositions were distributed across all groups with low to average annual incomes. Overall, the subjective RF-EMF exposure levels, health anxiety, and cancer risk perception in this cluster were low.

Cluster 2 (Anxious group) had more females and a higher proportion of middle-aged and older individuals. The basic demographics of Cluster 2 were males: 39.5%, age 20–29: 9.9%, age 30–39: 13.9%, age 40–49: 25.0%, age 50–59: 26.2%, and age over 60: 25.0%. There was a high proportion of individuals with a college degree or higher education level and those with education in RF-EMFs [24.2% (technical college) /53.3% (university, graduate school)]. The family compositions mostly consisted of more than two people, with a slightly high proportion of them having children aged under 15 years. Awareness levels of RF-EMFs, subjective RF-EMF exposure levels, health anxiety, and cancer risk perception were high in this cluster. The risk perception of other factors in daily life was also high.

Cluster 3 (Low-interest group) had more females and a higher proportion of middle-aged and older individuals. The basic demographics of Cluster 3 were males: 40.6%, age 20–29: 12.9%, age 30–39: 14.4%, age 40–49: 22.9%, age 50–59: 23.9%, and age over 60: 26.0%. There was a low proportion of individuals with a college degree or higher education level [23.2% (technical college) /48.9% (university, graduate school)]. Interest in new technologies (5G) was low, and they had a certain level of subjective RF-EMF exposure, with moderate health anxiety and cancer risk perception.

Cluster 4 (High-interest group) had a very high proportion of males distributed across all ages. The basic demographics of Cluster 4 were males: 79.3%, age 20–29: 19.2%, age 30–39: 16.6%, age 40–49: 20.0%, age 50–59: 23.2%, and age over 60: 21.1%. There was a high rate of individuals with a technical college degree or higher education level (64.7%) and those with education in RF-EMFs (13.8%). The family composition consisted of multiple members, with a high proportion including children under 15 years old, and the households tended to have higher annual incomes. This group had very high objective knowledge, but a certain level of subjective RF-EMF exposure, and answered with high confidence. Health anxiety and cancer risk perception were moderate.

### 3.4 Changes in attitudes toward RF-EMFs between RC and control groups in each cluster

#### 3.4.1 Changes in attitudes immediately after viewing the website (sub T1-T2)

Table 4 presents the dependence of risk communication on the cluster. Immediately after viewing the website (Sub T1-T2), there were fluctuations in attitudes toward RF-EMFs for several items. Data were expressed as median (25 percentile to 75 percentile) and “*r*” represents the effect size derived using *Z* score and sample size. Although changes from T1 occurred in several items for Clusters 1–3, no change was observed in Cluster 4 except for one item. Website viewing induced changes in the perception of health anxiety related to RF-EMFs but did not affect the carcinogenic risk perception associated with mobile phones.

**Table 4:** Changes in attitudes toward RF-EMFs determined from T1-T2 (Sub T1-T2) and T1-T3 (Sub T1-T3) differences among four clusters.

Cluster 1 (Non-anxious)	Sub T1-T2					Sub T1-T3				
Variable	Control (*n* = 108) median (25th percentile to 75th percentile)	RC group (*n* = 92) median (25th percentile to 75th percentile)	*p*	*r*	Diection of RC	Control (*n* = 108) median (25th percentile to 75th percentile)	RC group (*n* = 92) median (25th percentile to 75th percentile)	*p*	*r*	Diection of RC
Awareness of RF-EMFs in daily life	0.0 (−1.0 – 0.0)	0.0 (0.0 – 1.0)	**0.00**	0.21	↑	0.0 (−1.0 – 0.0)	0.0 (0.0 – 0.0)	0.12	0.11	–
Subjective RF-EMF exposure levels (sum of 8 sources)	0.0 (−3.0 – 2.0)	−1.0 (−7.0 – 3.0)	0.17	0.10	–	0.0 (−5.0 – 4.0)	−1.0 (−7.5 – 2.0)	**0.05**	0.14	↓
Confidence of subjective RF-EMF exposure levels (sum of 8 sources)	1.0 (0.0 – 8.0)	0.0 (0.0 – 8.0)	0.50	0.05		5.5 (0.0 – 8.0)	0.0 (0.0 – 8.0)	**0.00**	0.22	*
Concerns about adverse health effects of RF-EMF exposures (sum of 8 sources)	0.0 (0.0 – 1.0)	0.0 (0.0 – 8.0)	**0.00**	0.20	↑	0.0 (0.0 – 1.8)	0.0 (0.0 – 1.0)	0.80	0.02	–
Needs for RF-EMF usage activities (sum of 6 activities)	0.0 (−2.0 – 2.0)	0.0 (−2.0 – 2.0)	0.69	0.03	–	0.0 (−1.0 – 3.0)	0.0 (−1.0 – 2.5)	0.59	0.04	
Concerns about adverse health effects of RF-EMF usage activities (sum of 6 activities)	0.0 (0.0 – 1.0)	0.0 (0.0 – 6.0)	**0.01**	0.20	↑	0.0 (0.0 – 1.0)	0.0 (0.0 – 0.0)	0.23	0.08	
Awareness of the advantages of 5G	0.0 (0.0 – 1.0)	0.0 (0.0 – 0.5)	**0.02**	0.17	↓	0.0 (0.0 – 1.0)	0.0 (−1.0 – 1.0)	0.29	0.07	
Awareness of concerns about 5G	0.0 (0.0 – 0.0)	0.0 (0.0 – 0.0)	0.14	0.10	–	0.0 (0.0 – 0.0)	0.0 (0.0 – 0.0)	0.76	0.02	
Subjective knowledge	0.0 (0.0 – 0.0)	0.0 (0.0 – 1.0)	**0.00**	0.22	↑	0.0 (0.0 – 0.0)	0.0 (0.0 – 0.0)	0.12	0.11	
Carcinogenic risk perception (RF-EMFs: mobile phone)	0.0 (0.0 – 1.0)	0.0 (0.0 – 1.0)	0.60	0.04	–	0.0 (0.0 – 1.0)	0.0 (−1.0 – 1.0)	0.71	0.03	
Carcinogenic risk perception (ELF-EMFs: power lines)	0.0 (0.0 – 1.0)	0.0 (0.0 – 1.0)	0.81	0.02		0.0 (−1.0 – 1.0)	0.0 (0.0 – 1.0)	0.86	0.01	
Carcinogenic risk perception (sum of other 5 sources)	1.0 (0.0 – 4.0)	2.0 (0.0 – 4.0)	0.41	0.06		1.5 (−1.0 – 4.0)	1.0 (−1.0 – 4.0)	0.54	0.04	
Objective knowledge	−1.0 (−3.0 – 0.0)	0.0 (−2.0 – 1.0)	0.18	0.09		−2.0 (−4.0 – 0.0)	−1.0 (−4.0 – 0.0)	0.25	0.08	

Cluster 2 (Anxious)	Sub T1-T2					Sub T1-T3				
Variable	Control (*n* = 51) median (25th percentile to 75th percentile)	RC group (*n* = 56) median (25th percentile to 75th percentile)	*p*	*r*	Diection of RC	Control (*n* = 51) median (25th percentile to 75th percentile)	RC group (*n* = 56) median (25th percentile to 75th percentile)	*p*	*r*	Diection of RC
Awareness of RF-EMFs in daily life	0.0 (0.0 – 1.0)	0.0 (0.0 – 1.0)	0.60	0.05	–	0.0 (−1.0 – 1.0)	0.0 (0.0 – 1.0)	0.28	0.11	–
Subjective RF-EMF exposure levels (sum of 8 sources)	0.0 (−2.0 – 4.0)	−2.0 (−7.8 – 0.8)	**0.02**	0.23	↓	−1.0 (−5.0 – 3.0)	0.0 (−5.5 – 3.0)	0.67	0.04	
Confidence of subjective RF-EMF exposure levels (sum of 8 sources)	1.0 (0.0 – 8.0)	0.0 (−3.0 – 3.0)	**0.01**	0.24	↑	0.0 (−1.0 – 4.0)	0.0 (0.0 – 6.8)	0.52	0.06	
Concerns about adverse health effects of RF-EMF exposures (sum of 8 sources)	0.0 (−7.0 – 4.0)	−1.0 (−8.0 – 1.0)	0.12	0.15	–	0.0 (−4.0 – 1.0)	0.0 (−6.0 – 0.0)	0.38	0.08	
Needs for RF-EMF usage activities (sum of 6 activities)	0.0 (−2.0 – 2.0)	0.0 (−1.0 – 2.0)	0.76	0.03		0.0 (−2.0 – 1.0)	1.0 (0.0 – 3.0)	**0.01**	0.24	↑
Concerns about adverse health effects of RF-EMF usage activities (sum of 6 activities)	0.0 (−4.0 – 2.0)	−2.0 (−5.8 – 0.0)	**0.03**	0.22	↓	0.0 (−3.0 – 1.0)	−2.5 (−6.0 – 1.0)	0.10	0.16	–
Awareness of the advantages of 5G	0.0 (0.0 – 0.0)	0.0 (−1.0 – 0.0)	0.12	0.15	–	0.0 (0.0 – 0.0)	0.0 (−0.8 – 1.0)	0.80	0.03	
Awareness of concerns about 5G	0.0 (−1.0 – 0.0)	0.0 (−1.0 – 0.0)	0.38	0.09		0.0 (−1.0 – 1.0)	0.0 (−1.0 – 0.0)	0.57	0.06	
Subjective knowledge	0.0 (0.0 – 0.0)	0.0 (0.0 – 1.0)	**0.03**	0.21	↑	0.0 (0.0 – 0.0)	0.0 (0.0 – 0.0)	0.59	0.05	
Carcinogenic risk perception (RF-EMFs: mobile phone)	0.0 (−1.0 – 0.0)	0.0 (−2.0 – 0.0)	0.43	0.08	–	0.0 (−1.0 – 0.0)	0.0 (−1.0 – 1.0)	0.42	0.08	
Carcinogenic risk perception (ELF-EMFs: power lines)	0.0 (−1.0 – 0.0)	0.0 (−1.0 – 0.0)	0.46	0.07		0.0 (−1.0 – 0.0)	0.0 (−1.0 – 0.0)	0.84	0.02	
Carcinogenic risk perception (sum of other 5 sources)	1.0 (−2.0 – 3.0)	0.5 (−2.0 – 2.8)	0.93	0.01		0.0 (−2.0 – 2.0)	0.0 (−2.0 – 4.0)	0.71	0.04	
Objective knowledge	−1.0 (−2.0 – 0.0)	−1.0 (−2.0 – 1.0)	0.32	0.10		−2.0 (−3.0 – 0.0)	−2.0 (−3.0 – 0.0)	0.89	0.01	

Cluster 3 (Low-interest)	Sub T1-T2					Sub T1-T3				
Variable	Control (*n* = 153) median (25th percentile to 75th percentile)	RC group (*n* = 160) median (25th percentile to 75th percentile)	*p*	*r*	Diection of RC	Control (*n* = 153) median (25th percentile to 75th percentile)	RC group (*n* = 160) median (25th percentile to 75th percentile)	*p*	*r*	Diection of RC
Awareness of RF-EMFs in daily life	0.0 (0.0 – 0.0)	0.0 (0.0 – 1.0)	0.13	0.09	–	0.0 (0.0 – 1.0)	0.0 (−1.0 – 0.0)	0.08	0.08	–
Subjective RF-EMF exposure levels (sum of 8 sources)	0.0 (−3.0 – 4.0)	0.0 (−5.0 – 3.0)	0.28	0.06		0.0 (−4.0 – 4.0)	0.0 (−4.0 – 3.8)	0.02		0.02
Confidence of subjective RF-EMF exposure levels (sum of 8 sources)	0.0 (0.0 – 5.0)	0.0 (0.0 – 8.0)	**0.01**	0.15	↑	0.0 (0.0 – 8.0)	0.0 (0.0 – 8.0)	0.02		0.02
Concerns about adverse health effects of RF-EMF exposures (sum of 8 sources)	0.0 (−2.0 – 2.0)	0.0 (0.0 – 3.0)	0.13	0.09	–	0.0 (−1.0 – 3.0)	0.0 (−0.8 – 1.0)	0.03		0.03
Needs for RF-EMF usage activities (sum of 6 activities)	0.0 (−1.0 – 2.0)	0.0 (−3.0 – 1.0)	**0.01**	0.16	↓	0.0 (−1.0 – 2.0)	1.0 (−1.0 – 2.8)	0.09		0.09
Concerns about adverse health effects of RF-EMF usage activities (sum of 6 activities)	0.0 (−1.0 – 1.0)	0.0 (0.0 – 1.0)	0.34	0.05	–	0.0 (−1.0 – 1.0)	0.0 (−1.0 – 0.8)	0.04		0.04
Awareness of the advantages of 5G	0.0 (0.0 – 1.0)	0.0 (0.0 – 0.8)	0.17	0.08	–	0.0 (0.0 – 1.0)	0.0 (0.0 – 0.0)	0.01		0.01
Awareness of concerns about 5G	0.0 (0.0 – 0.0)	0.0 (0.0 – 0.0)	0.25	0.06	–	0.0 (0.0 – 0.0)	0.0 (0.0 – 0.0)	0.03		0.03
Subjective knowledge	0.0 (0.0 – 0.0)	0.0 (0.0 – 0.0)	0.11	0.09	–	0.0 (0.0 – 0.0)	0.0 (0.0 – 0.0)	0.04		0.04
Carcinogenic risk perception (RF-EMFs: mobile phone)	0.0 (−1.0 – 0.0)	0.0 (−1.0 – 1.0)	0.56	0.03	–	0.0 (−1.0 – 1.0)	0.0 (−0.8 – 1.0)	0.04		0.07
Carcinogenic risk perception (ELF-EMFs: power lines)	0.0 (−1.0 – 0.0)	0.0 (−1.0 – 0.0)	0.45	0.04	–	0.0 (−1.0 – 0.0)	0.0 (−1.0 – 0.0)	0.01		0.04
Carcinogenic risk perception (sum of other 5 sources)	0.0 (−2.0 – 2.0)	0.0 (−2.0 – 2.0)	0.38	0.05	–	0.0 (−2.0 – 2.0)	0.0 (−2.0 – 2.0)	0.04		0.01
Objective knowledge	−1.0 (−3.0 – 0.0)	−1.0 (−2.0 – 1.0)	**0.01**	0.15	↑	−2.0 (−4.0 – 0.0)	−1.0 (−3.0 – 0.0)	0.07		0.04

Cluster 4 (High-interest)	Sub T1-T2					Sub T1-T3				
Variable	Control (*n* = 78) median (25th percentile to 75th percentile)	RC group (*n* = 71) median (25th percentile to 75th percentile)	*p*	*r*	Diection of RC	Control (*n* = 78) median (25th percentile to 75th percentile)	RC group (*n* = 71) median (25th percentile to 75th percentile)	*p*	*r*	Diection of RC
Awareness of RF-EMFs in daily life	0.0 (−1.0 – 0.0)	0.0 (−1.0 – 0.0)	0.81	0.02	–	0.0 (−1.0 – 0.0)	0.0 (−1.0 – 0.0)	0.39	0.07	–
Subjective RF-EMF exposure levels (sum of 8 sources)	−1.5 (−7.0 – 2.0)	−3.0 (−6.0 – 2.0)	0.80	0.02		−2.0 (−6.0 – 2.0)	−3.0 (−6.0 – 1.0)	0.88	0.01	
Confidence of subjective RF-EMF exposure levels (sum of 8 sources)	0.0 (−6.3 – 1.0)	0.0 (−8.0 – 3.0)	0.95	0.01		−0.5 (−6.0 – 0.0)	0.0 (−4.0 – 2.0)	0.33	0.08	
Concerns about adverse health effects of RF-EMF exposures (sum of 8 sources)	0.0 (−2.0 – 1.0)	0.0 (−2.0 – 1.0)	0.46	0.06		0.0 (−2.0 – 2.0)	0.0 (−1.0 – 1.0)	0.53	0.05	
Needs for RF-EMF usage activities (sum of 6 activities)	0.0 (−1.3 – 2.0)	0.0 (−3.0 – 1.0)	0.11	0.13		0.0 (−2.0 – 2.0)	0.0 (−1.0 – 1.0)	0.33	0.08	
Concerns about adverse health effects of RF-EMF usage activities (sum of 6 activities)	0.0 (−3.3 – 1.0)	0.0 (−1.0 – 1.0)	0.19	0.11		0.0 (−1.0 – 1.0)	0.0 (−2.0 – 1.0)	0.71	0.03	
Awareness of the advantages of 5G	0.0 (0.0 – 1.0)	0.0 (−1.0 – 0.0)	0.29	0.09		0.0 (0.0 – 1.0)	0.0 (0.0 – 1.0)	0.90	0.01	
Awareness of concerns about 5G	0.0 (0.0 – 0.0)	0.0 (0.0 – 0.0)	0.43	0.07		0.0 (0.0 – 0.0)	0.0 (0.0 – 0.0)	0.31	0.08	
Subjective knowledge	0.0 (−1.0 – 0.0)	0.0 (0.0 – 0.0)	**0.02**	0.20	↑	0.0 (−1.0 – 0.0)	0.0 (0.0 – 0.0)	0.52	0.05	
Carcinogenic risk perception (RF-EMFs: mobile phone)	0.0 (0.0 – 1.0)	0.0 (−1.0 – 1.0)	0.14	0.12	–	0.0 (−1.0 – 0.3)	0.0 (0.0 – 1.0)	0.44	0.06	
Carcinogenic risk perception (ELF-EMFs: power lines)	0.0 (−1.0 – 1.0)	0.0 (−1.0 – 0.0)	0.42	0.07		0.0 (−1.0 – 1.0)	0.0 (−1.0 – 1.0)	0.88	0.01	
Carcinogenic risk perception (sum of other 5 sources)	0.0 (−2.0 – 2.3)	1.0 (−1.0 – 3.0)	0.07	0.15		0.0 (−1.0 – 3.0)	1.0 (−1.0 – 3.0)	0.96	0.00	
Objective knowledge	−1.0 (−3.0 – 0.0)	−1.0 (−3.0 – 0.0)	0.57	0.05		−2.0 (−3.3 – 0.0)	−2.0 (−4.0 – −1.0)	0.49	0.06	

The Mann–Whitney U-test was applied to the analysis. R, effect size; Bold, statistically significant. ↑ Represents the increased effect and ↓ Represents the decreased effect. The “-” represents no statistical change between RC and control group.

**Figure 2:**
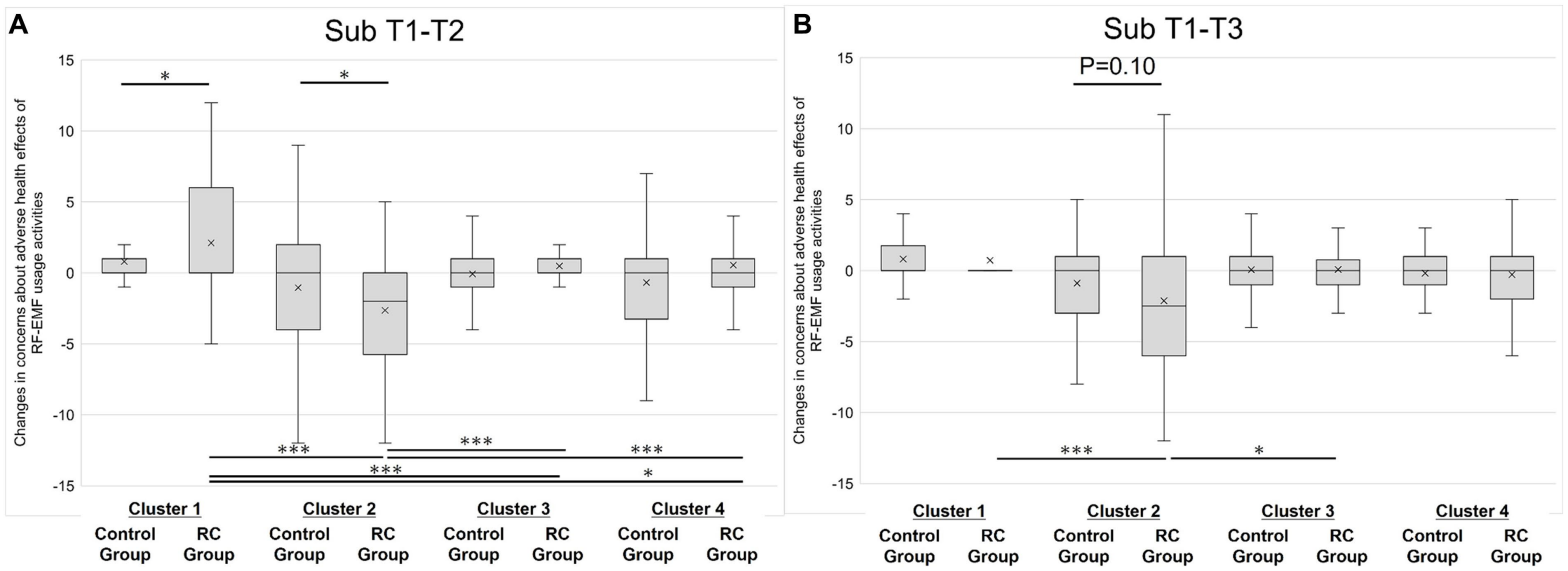
Changes in concerns about adverse health effects of RF-EMF usage activities (sum of 6 activities) for panel **(A)**, T1-T2 (Sub T1-T2); for panel **(B)**, T1-T3 (Sub T1-T3). *p < 0.05 and ***p < 0.001.

In Cluster 1 (Non-anxious group), immediately after website viewing (Sub T1-T2), there was a temporary significant increase in the level of awareness of RF-EMFs (*p* < 0.001, *r* = 0.21, Mann–Whitney U-test). In addition, there was a negative change in attitudes toward RF-EMFs, including increased concerns about the adverse health effects of RF-EMF exposure (sum of eight sources) and concerns about the adverse health effects of RF-EMF usage activities (Figure 2A), and a decrease in the recognition of the advantages of 5G (*p* < 0.001, *p* = 0.01 and 0.02, *r* = 0.20, 0.20, and 0.17, Mann–Whitney U-test), but all returned to the same level as baseline levels in T1 5 weeks after website viewing. Subjective knowledge increased owing to website viewing (*p* < 0.01 *r* = 0.22, Mann–Whitney U-test).

In Cluster 2 (Anxious group), the subjective RF-EMF exposure level decreased immediately after website viewing (*p* = 0.02, *r* = 0.23, Mann–Whitney U test), whereas confidence decreased simultaneously (*p* = 0.01, *r* = 0.24, Mann–Whitney U-test). Concerns about the adverse health effects of RF-EMF usage activities decreased immediately after viewing (Figure 2A, *p* = 0.03, *r* = 0.22, Mann–Whitney U-test). Subjective knowledge increased owing to website viewing, as in Cluster 1 (Non-anxious group) (*p* = 0.03, *r* = 0.21, Mann–Whitney U-test).

In Cluster 3 (Low-interest group), although there was no fluctuation in subjective RF-EMF exposure level, confidence significantly increased (*p* = 0.01, *r* = 0.15, Mann–Whitney U-test). However, similarly to Cluster 1 (Non-anxious group), there was a negative change in attitudes toward RF-EMFs, and the need for RF-EMF usage activities temporarily decreased (*p* = 0.01, *r* = 0.16, Mann–Whitney U-test). As for objective knowledge, the results showed that the control group showed a decrease in the number of correct answers as participants were unable to answer questions related to website viewing, but the degree of decrease in the number of correct answers was slight in the RC group compared with the control group (*p* = 0.01, *r* = 0.15, Mann–Whitney U-test).

In Cluster 4 (High-interest group), no statistically significant changes in attitudes toward RF-EMFs were observed, except for subjective knowledge (*p* = 0.02, *r* = 0.20, Mann–Whitney U-test).

For intercluster differences in T1-T2 for concerns about RF-EMF usage activities, differences were observed between some clusters in T1-T2 (Figure 2A). In the RC group of Cluster 1, where an increase in scores (increase in anxiety) was observed, a statistically significant difference was noted compared with all other RC groups (Clusters 2, 3, and 4). Similarly, in the RC group of Cluster 2, where a decrease in scores (decrease in anxiety) was observed, a statistically significant difference was noted compared with all other RC groups (Clusters 1, 3, and 4).

#### 3.4.2 Changes in attitudes 5 weeks after viewing the website (sub T1-T3)

Overall, only three changes were observed in attitudes toward RF-EMFs 5 weeks after website viewing (Table 4). Data were expressed as median (25 percentile to 75 percentile), and “*r*” represents the effect size derived using *Z* score and sample size.

In Cluster 1 (Non-anxious group), 5 weeks after website viewing, the subjective RF-EMF exposure levels were decreased in the Follow-up RC group (*p* = 0.05, *r* = 0.15, Mann–Whitney U-test). Interestingly, the control group showed an increase in confidence in subjective RF-EMF exposure levels (*p* < 0.001, *r* = 0.22, Mann–Whitney U-test). In Cluster 2 (Anxious group), the need for RF-EMF usage activities increased in the RC group 5 weeks after website viewing (*p* = 0.01, *r* = 0.24, Mann–Whitney U-test).

In Cluster 2, a decreasing trend for concerns remained even 5 weeks after website viewing (Figure 2B, *p* = 0.10, *r* = 0.16). Because of this, for intercluster differences in T1-T3 for concerns about RF-EMF usage activities, there were significant changes between clusters 1 and 2, and between 2 and 3 in the Follow-up RC group (Figure 2B). In Cluster 3 (Low-interest group), the differences in objective knowledge observed in T2 was diminished in T3.

#### 3.4.3 Subanalysis by gender and age in sub T1-T2

**Figure 3:**
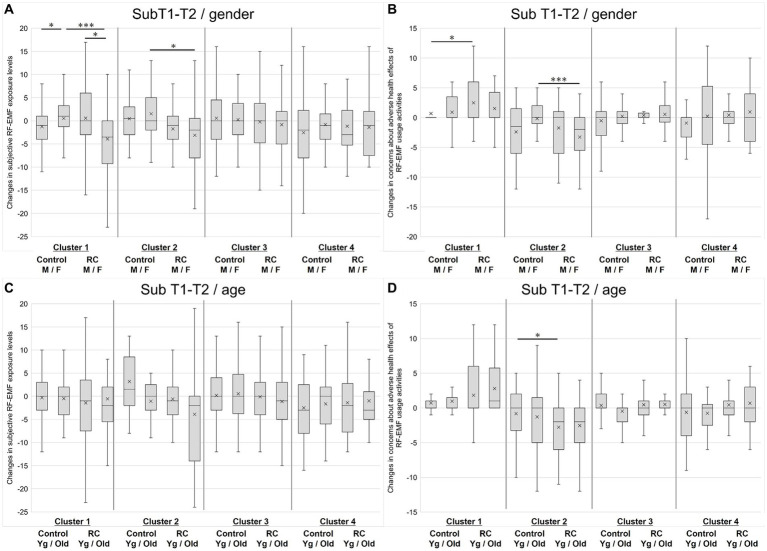
Sub-analysis by gender and age. **(A)** Changes in subjective RF-EMF levels in T1-T2 stratified by gender and cluster. **(B)** Changes in concerns about RF-usage activities in T1-T2 stratified by gender and cluster. **(C)** Changes in subjective RF-EMF levels in T1-T2 stratified by age and cluster. **(D)** Changes in concerns about RF-usage activities in T1-T2 stratified by age and cluster. M, male; F, female; Yg, younger generation (age 20–49); Old, older generation (age > =50). *p < 0.05 and ***p < 0.001.

Figure 3 shows the dependence of gender and age on the effects of risk communication. Changes in subjective RF-EMF exposure levels and concerns about adverse health effects of RF-EMF usage activities in T2 (sub T1-T2) were analyzed. The results showed significant changes more often in gender than in age. Data were expressed as median (25 percentile to 75 percentile) and “r” represents the effect size derived using Z score and sample size.

Figure 3A shows the dependence of gender on subjective RF-EMF exposure levels. In Cluster 1 (Non-anxious group), females had higher perceived exposure levels than males in the control group. Significant differences were observed between males and females in the control group [expressed as median (25 percentile to 75 percentile): male, −1.0 (−4.0–1.0); female, 1.0 (−1.3–3.3); *p* = 0.01, *r* = 0.24, Mann–Whitney U-test]. For gender comparison in the RC group, females showed a marked decrease in the score [male, 0.0 (−3.0–6.0); female, −3.5 (0.0 – −9.3); *p* = 0.01, *r* = 0.25, Mann–Whitney U-test]. Females in the RC group showed significantly lower perceived exposure levels after the risk communication compared with those in the control group (*p* < 0.01, 0.36, Mann–Whitney U-test).

In Cluster 2 (Anxious group), females in the RC group showed significantly lower perceived exposure levels than those in the control group [RC group, −2.0 (−8.0 – 0.5); control group, 0.0 (−2.0 – 5.0); *p* = 0.05, *r* = 0.25, Mann–Whitney U-test]. For males, there was no significant difference, but there was a trend toward a decrease in the RC group [RC group, −1.0 (−4.0 – 1.0); control group, 0.5 (−3.0 to 3.0); *p* = 0.15, *r* = 0.22, Mann–Whitney U-test]. No clear gender effects were observed in Clusters 3 and 4.

Figure 3B shows the dependence of gender on concerns about adverse health effects of RF-EMF usage activities. Similar to subjective RF-EMF exposure levels, in Cluster 1, females had higher anxiety levels than males in the control group. A significant difference was detected between the male respondents in the RC group and the control group, with scores of 0.0 (0.0 – 0.0) for the RC group and 0.0 (0.0 – 3.5) for the control group; *p* < 0.01, *r* = 0.27, Mann–Whitney U-test, indicating that anxiety increased in the RC group. In contrast, in Cluster 2, female respondents who received the risk communication showed significantly reduced anxiety levels, with scores of −2.0 (−5.5 – 0.0) for the RC group and 0.0 (−1.0 – 2.0) for the control group; *p* < 0.001, *r* = 0.45, Mann–Whitney U-test.

Regarding the age dependence of the changes in subjective RF-EMF exposure levels upon risk communication (Figure 3C), in Clusters 2 and 3, there was an increasing trend in the RC group regardless of age, but no clear age effects were observed.

Regarding concerns about adverse health effects of RF-EMF usage activities after the risk communication (Figure 3D), in Cluster 2, younger respondents who received the risk communication showed significantly lower anxiety levels than younger respondents in the control group, with scores of −2.0 (−6.0 – 0.0) for the RC group and 0.0 (−3.3 – 2.0) for the control group; *p* = 0.03, *r* = 0.31, Mann–Whitney U-test. A similar trend was observed in Cluster 3 (Low-interest group), but no significant difference was detected.

### 3.5 Analysis of objective knowledge

Since Q1–Q5 were questions regarding objective knowledge of general RF-EMFs in T2 and T3, and Q6–Q10 were related to the contents of the risk communication website, the understandability of the website and the retention of the knowledge were assessed. The RC group showed a significantly greater number of correct answers to Q6–Q10 in T2 (*p* < 0.001, *r* = 0.12, Mann–Whitney U-test), indicating that the RC group viewed the website and obtained certain levels of knowledge. The numbers of correct answers for Q6-Q10 were as follows, expressed as median (25 percentile to 75 percentile): RC group, 2.0 (1.0–4.0); control group, 2.0 (0.0–3.0). The number of “no idea” answers for Q6–Q10 was significantly decreased in the RC group in T2 (*p* < 0.001, *r* = 0.12, Mann–Whitney U-test). The numbers of “no idea” answers of the groups were as follows, expressed as median (25 percentile to 75 percentile): RC group, 1.0 (0.0–4.0); control group, 2.0 (0.05.0).

However, there were no significant differences in the numbers of correct answers for Q1–Q5 (both in T2 and T3) and for Q6–Q10 in T3. Moreover, there were no significant differences in the numbers of “no idea” answers for Q1–Q5 (both in T2 and T3) and Q6–Q10 in T3.

## 4 Discussion

In this study, we first classified the general public’s attitudes toward RF-EMFs based on 12 items related to RF-EMF perception except for objective knowledge (Table 3). As a result, it was possible to classify them on the basis of the presence or absence of anxiety about RF-EMFs and the level of interest. The clusters were named Cluster 1 (Non-anxious group), Cluster 2 (Anxious group), Cluster 3 (Low-interest group), and Cluster 4 (High-interest group). Furthermore, we evaluated the risk communication of RF-EMFs via website viewing by each cluster, which showed that the clusters exhibited different responses (Table 4). Although some items tended to show changes that were maintained 5 weeks after website viewing, most items showed that the impact of website viewing disappeared after 5 weeks.

Among the 12 items related to attitudes toward RF-EMFs, the following five items are considered related to the evaluation of risk communication: subjective RF-EMF exposure level, concerns about adverse health effects of RF-EMF exposure (8 sources), concerns about the adverse health effects of RF-EMF usage activities (sum of 6 activities), awareness of concerns about 5G, and carcinogenic risk perception (RF-EMFs: mobile phones). Regarding these five items, a decrease in the subjective RF-EMF exposure level was observed immediately after website viewing (Sub T1-T2) in the RC group (*p* = 0.03, *r* = 0.11), but the effect disappeared 5 weeks after viewing (Sub T1-T3) (Table 2). Analyzing the responses by cluster, temporary increases (Cluster 1: *p* = 0.01, *r* = 0.20) and decreases (Cluster 2: *p* = 0.03, *r* = 0.22) in concerns about adverse health effects of RF-EMF usage activities were observed. The increase in anxiety in Cluster 1 disappeared after 5 weeks, but the extent of reduction in anxiety in Cluster 2 showed a decreasing trend (*p* = 0.10, *r* = 0.16) (Table 4). This suggests that the website used in this study functioned as a form of risk communication and was particularly effective for Cluster 2 (Anxious group). Since no changes were observed in Cluster 4 except for subjective knowledge, it can be considered that this cluster is less responsive to risk communication.

The results of the subgroup analysis showed significant changes more often in gender than in age. The gender differences were especially obvious in Clusters 1 and, where female respondents tended to perceive higher subjective RF-EMF exposure levels and anxiety related to RF-EMF usage activities. Several groups have reported factors related to the risk perception of EMFs or RF-EMFs (29–31). In particular, Kim et al. reported that among those with a high-risk perception of RF-EMFs from mobile phones, women showed a higher risk perception than men, and individuals with more personal knowledge about RF-EMFs had a higher risk perception (31). The result of the present study, especially the observations in Clusters 1 and 2, aligns with those of previous studies. In contrast, in this study, only a few modifications by age were observed. Since we used only two categories for gender due to the small sample size in the present study, a more detailed classification of age categories might reveal age differences.

The temporary fluctuations in concerns about the adverse health effects of RF-EMF usage activities observed in Clusters 1 and 2 could be caused by the evaluation scale used in the present study. The purpose of this study was to clarify the extent to which changes in attitudes toward RF-EMFs occurred in each cluster after viewing the website for risk communication; hence, the evaluation scale for differences between T1-T2 and T1-T3 was used. The raw scores for this item are the sum of the answers to six items on a four-point scale, distributed between 6 and 24 points. The median (25 percentile–75 percentile) baseline score (T1) for Cluster 1 was 6 (6, 7) and that for Cluster 2 was 18 (15–19) (Table 3), which were very low and high, respectively, possibly making it easier to detect fluctuations. In other words, the perception of health anxiety in these clusters may be highly susceptible to fluctuations.

The risk communication conducted in this study temporarily increased anxiety about RF-EMFs (Cluster 1) and reduced the need for RF-EMF usage activities (Clusters 1 and 3) and the awareness of the advantages of 5G (Cluster 1). These results indicate that negative perceptions of RF-EMFs occur through risk communication. In previous studies, it was shown that risk communication regarding precautionary measures against RF-EMFs increases risk perception (9–12). Furthermore, Freudenstein et al. showed that three patterns of EMF risk communication (G1: IARC’s press release on the carcinogenicity of mobile phones; G2: G1 + textual explanation; and G3: emphasis on the fact that carcinogenic risk is only related to mobile phones) showed increased risk perception toward all sources of EMFs in all experimental groups (32). MacGregor et al. also reported that the specific content of leaflets strongly influenced the belief in the harmfulness of EMFs (33). Considering these prior studies, conducting the risk communication about EMFs, including RF-EMFs, has a certain effect on inducing anxiety and risk perception, and the temporary negative perception toward RF-EMFs observed in this study was natural. Freudenstein et al. (32) showed an increased risk perception toward all sources of electromagnetic fields, as in the results of this study. The present study revealed no clear difference in carcinogenic risk perception between mobile phones (RF-EMFs) and power lines (ELF-EMFs).

Regarding methods of risk communication that do not elicit anxiety in the non-anxious population, it is generally reported that tailored communication (including tailored risk communication) to the individual is highly effective (34–38). Therefore, preparing multiple types of information and providing them in accordance with an individual’s level of anxiety may be one way to avoid unnecessary anxiety. Additionally, there are reports recommending that after one-way risk communication via a website, two-way risk communication in the presence of experts should be conducted (38). To minimize the elicitation of anxiety in certain populations as much as possible, personalized risk communication and two-way risk communication following website viewing may be effective.

In the present study, we observed no detectable changes in carcinogenic risk perception of RF-EMFs. This could be an influence of content evaluation. The results of previous studies on the impact of uncertainty in the risk perception of electromagnetic fields have suggested that qualitative expressions of uncertainty in the hazard identification of electromagnetic fields reduced confidence in professional competence, but quantitative descriptions (uncertainty about the degree of risk) did not affect risk perception (39). The explanation of the adverse health effects of RF-EMFs on the website used in this study was that “there have been no reproducible reports of carcinogenicity from mobile phones to date.” If viewers perceived uncertainty in hazard identification from this statement, it is possible that sufficient trust in the website was not established and hence, risk perception did not decrease.

In this study, we investigated the attributes and social factors related to attitudes toward RF-EMFs, including age, gender, employment status, educational level, special education history regarding RF-EMFs, family situation, and income. Although we conducted subanalyses on age and gender, it is known that various factors are involved in the risk perception of EMFs (including RF-EMFs). For example, Mgbe et al. reported a significant positive correlation between the perception of EMF (including RF-EMF) risks and academic year among Nigerian students, suggesting a relationship between the period of education and perception of RF-EMF risks (40). Similarly, in our study, Cluster 2, in which health anxiety and cancer risk perception were high, showed that although the educational level of respondents was average, the proportion of those with special education history regarding RF-EMFs was high.

Moreover, similar to the effects of educational level, as our website provides information about exposure levels and knowledge regarding RF-EMFs, the impact of objective knowledge on risk perception is also an important consideration. Regarding objective and subjective knowledge, Pradhan et al., in a study conducted in India, reported that a lack of knowledge about mobile communication technology significantly impacted Indians’ risk perception, highlighting the necessity of risk communication (41). Seo et al. reported that individuals with knowledge of RF-EMFs had a higher risk perception (42). In our results, respondents with high levels of objective knowledge about RF-EMFs were in Cluster 4, but Cluster 2 also showed slightly higher correct response rates than the average. To explain this result, a subanalysis of the highly knowledgeable group (with a correct response rate of 80–100%) was conducted, and the results showed little change in concerns about adverse health effects of RF-EMF usage activities [0.0 (0.0 – 0.0), represented as median (25 percentile to 75 percentile)]. This implies that our findings did not align with those of previous research in terms of the knowledge of RF-EMFs. In the future, examining the reclassification of the clusters in T2 and T3 and changes from T1 will provide more accurate information regarding the impact of knowledge acquisition on risk perception.

Furthermore, previous research studies on EMF risk perception revealed various psychological factors (9, 29–33, 43–45) and multifactorial effects (29). They revealed that the general public evaluates the risks of RF-EMFs using various scales, such as emotional and moral assessments, perceived exposure (44), and factors originating from the source of exposure (e.g., frequency, intensity, and duration) (43, 45). Among the latter studies, Wiedemann et al., in addition to the traditional focus on measuring the magnitude of risk perception, conducted an expanded survey on “thematic relevance” (how often people think about a risk issue) and “discursive relevance” (how often people think about or discuss a risk issue) (29). Using thematic relevance, they distinguished between participants who were “enduringly concerned” and those who were not. They reported that enduringly concerned subjects perceived RF-EMF exposure as a moral and emotional issue and felt highly exposed to RF-EMFs, indicating the necessity for EMF risk analysis that considers multifactorial effects. Wilson et al. also suggested that risk perception is multidimensional and that a general unidimensional risk measure might accurately capture the perception of the severity of the consequences and the discrete emotions felt in response to those potential consequences (46). In our study, we evaluated the effect of risk communication on the basis of a unidimensional attitude toward RF-EMFs, and the lack of questions regarding other psychological factors is considered a limitation. However, considering these previous studies, we plan to conduct a more detailed analysis of cognitive changes in T1-T3 (such as reclassification of clusters and cross-lagged models) and incorporate psychological factors reported in previous studies (e.g., sense of self-protection and trust) into our future surveys.

Although there are some prior studies on risk perception and risk communication regarding the health effects of RF-EMFs, in this study, we classified the targets of risk communication on the basis of attitudes toward RF-EMFs, following up on the responses by cluster up to 5 weeks later. Such a study has not been conducted in previous research on risk communication regarding RF-EMFs; thus, this study provides some new findings for future risk communication strategies.

However, the following are the limitations of this study: sample bias, response acquisition methods, study duration, questionnaire validity and reliability, susceptibility to external factors (information collection), generalizability of results, and need for website evaluation:

Regarding sample bias, it is possible that the participants in the online survey do not fully represent the general population. This is because the survey title and purpose were clearly stated, potentially attracting participants with a special interest or prior knowledge about RF-EMFs, which may have influenced their responses. Additionally, online survey participants might be more proactive in using the internet. This limitation could be mitigated by using mail surveys in addition to web surveys.

Concerning the response acquisition methods, the data collected is based on self-reporting, which could introduce recall bias or social desirability bias. Furthermore, since qualitative data regarding attitudes toward RF-EMFs were not obtained in this study, the depth of understanding of the participants’ perceptions and attitudes is limited. Conducting open-ended comments and interviews would provide insights into participants’ underlying motivations and concerns.

Regarding the study duration, the survey was in total 10 weeks, with a follow-up period of 5 weeks. This duration was set exploratively, considering the lack of research on the persistence of the impact of RF-EMF risk communication on awareness, making the T2–T3 period 5 weeks. Most cognitive changes disappeared within 5 weeks, but it is necessary to confirm the effect and check for delayed effects. We have also collected data 3 months after the risk communication, which may provide a more comprehensive view upon further analysis.

Confirmation of the validity and reliability is one of the limitations of the present study. The results of the internal consistency in terms of anxiety were good or sufficient (Cronbach’s alpha >0.6) except for factor 4 (Benefit recognition). The intrarater reliability was also sufficient. However, items for benefit recognition must be modified using the results of reliability analysis. Since we created the questionnaire in an exploratory manner, further study to improve the questionnaire on a psychological basis is required. For example, cluster reclassification in T2 and T3 will provide insight for new questionnaire development. Moreover, in this study, cluster classification was based on anxiety and interest. However, since these factors may be interrelated, we will examine the independence of these factors in future investigations.

The inability to completely control external factors, such as participants’ collection of RF-EMF information, is another limitation. Such information collection might have influenced participants’ perceptions and attitudes, potentially confounding the effects of the risk communication website. We have investigated the opportunities for RF-EMF information collection among respondents during T1-T2 and T2-T3, and future analyses considering these factors will clarify this effect.

Regarding the generalizability of results, potential sample bias may mean that the findings cannot be generalized to other populations or situations. Additionally, as this study was conducted in Japan, cultural commonalities should be considered before applying these findings to other countries. Furthermore, further analysis for specific populations (e.g., high health literacy, pregnant women, etc.) will provide more insights for audience segmentation.

Finally, another limitation is that the impact of the website has not yet been evaluated. Although we provided minimum content through screenshots and then proceeded to responses to control the minimum information provided, the time and content of website browsing were left to the respondents. Therefore, the understanding and retention of the information provided through the website may vary among participants. The analysis of objective knowledge indicated a certain level of the impact of website viewing on the RC group; however, the retention of knowledge was very poor. Further analysis to estimate the impact of the website accurately is necessary. Moreover, although the content has been checked by experts, the design has not been reviewed by specialists, so the impact of the design on the results should also be considered.

For further studies, re-examining the T1-T2 or T2-T3 cluster reclassification or the changes in respondents’ psychology over time will provide more information about RF-EMF risk communication. It may also be necessary to investigate what kind of RF-EMF communication is appropriate for Cluster 4 identified in this study.

## 5 Conclusion

We studied the risk communication of RF-EMFs through website viewing by conducting three follow-up surveys over 10 weeks with the same participants. When classifying respondents into four clusters, characteristic fluctuations in attitudes toward RF-EMFs after website viewing were observed for each cluster. Specifically, Cluster 2 (Anxious group) was found to have a high level of understanding of the website and the outcome was directly linked to risk communication (reduction in concerns about the adverse health effects of RF-EMF usage activities). In this study, we demonstrated that providing objective and comprehensible information through a website can significantly reduce the perception of risk and concerns about the adverse health effects associated with RF-EMFs. This underscores the importance of transparency and education regarding RF-EMFs in risk communication to improve public awareness and alleviate unfounded fears.

## Data Availability

The raw data supporting the conclusions of this article will be made available by the authors, without undue reservation.

## Supplementary material

Supplementary Figure 1 Distribution of clusters in T1 to T3. **(A)** Control group. **(B)** RC group. The chi-square test was applied to the analysis.
