# Supplementary figures and images for: Effects of website-based risk communication of radio-frequency electromagnetic fields on general public
Source: Front Public Health. 2024 Sep 4;12:1438986. doi: 10.3389/fpubh.2024.1438986 (PMC11408325; doi:10.3389/fpubh.2024.1438986)

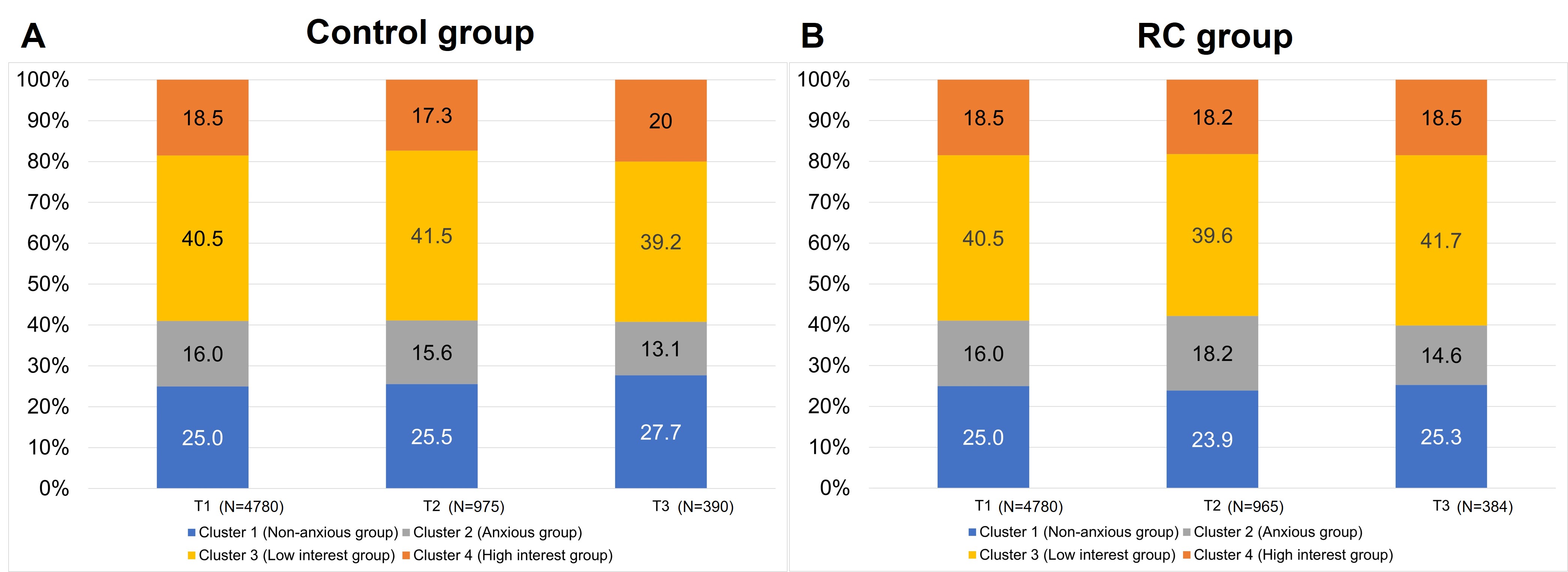

Supplement: Supplementary Figure 1 — Distribution of clusters in T1 to T3. (A) Control group. (B) RC group. The chi-square test was applied to the analysis. [file Image_1.JPEG]
